# Recognition of the neurobiological insults imposed by complex trauma and the implications for psychotherapeutic interventions^†^

**DOI:** 10.1192/pb.bp.114.047134

**Published:** 2015-04

**Authors:** Frank M. Corrigan, Alastair M. Hull

**Affiliations:** 1 Argyll & Bute Hospital, Lochgilphead, UK; 2 Perth Royal Infirmary, Perth, UK

## Abstract

Considerable research has been conducted on particular approaches to the psychotherapy of post-traumatic stress disorder (PTSD). However, the evidence indicates that modalities tested in randomised controlled trials (RCTs) are far from 100% applicable and effective and the RCT model itself is inadequate for evaluating treatments of conditions with complex presentations and frequently multiple comorbidities. Evidence at levels 2 and 3 cannot be ignored. Expert-led interventions consistent with the emerging understanding of affective neuroscience are needed and not the unthinking application of a dominant therapeutic paradigm with evidence for PTSD but not complex PTSD. The over-optimistic claims for the effectiveness of cognitive–behavioural therapy (CBT) and misrepresentation of other approaches do not best serve a group of patients greatly in need of help; excluding individuals with such disorders as untreatable or treatment-resistant when viable alternatives exist is not acceptable.

The healing of emotional wounds is an endogenous, dynamic process which, fortunately, occurs spontaneously in most people whose traumatic experiences resolve over time. Just as the pain of grief diminishes as the body feelings become less insistent and perspectives change, so other adverse events can be processed naturally and emotional healing can occur. When a bereavement is traumatic, for whatever reason, the natural healing process may be blocked and the pain fails to resolve. This is echoed after other adversity: the distress and its associated body feeling, however subtle, continue to intrude on awareness and impair the capacity for positive affects. A failure to recognise the intrinsic homeostatic, emotional healing potential of the brain/mind leads to an overemphasis on techniques for regulation of distress and a denial of opportunity for resolution.

## Simple protocols for PTSD, complex post-traumatic presentations and help-seeking

There are interventions available to treat established post-traumatic stress disorder (PTSD) that have been shown to be effective. Reviews of the literature and expert consensus have supported the use of trauma-focused cognitive–behavioural therapy (TF-CBT; especially treatment programmes involving imaginal and *in vivo* exposure), and eye movement desensitisation and reprocessing (EMDR).^1,2^ Moreover, the National Institute for Health and Care Excellence (NICE) guidelines on PTSD recommend that all individuals with PTSD should be offered TF-CBT or EMDR.^3^ However, psychotherapy studies for PTSD typically exclude for comorbidity and complexity and do not report adverse effects: it should not be assumed that therapy is either positive or, at worst, neutral. The wrong therapy, by the wrong therapist or with the wrong timing (or a combination thereof) may be psychonoxious. Reviews and guidelines present the evidence base for PTSD, but rarely overtly identify the dichotomy between the patients seen in research studies and those with the range of clinical presentations encountered after traumatic experiences, even though the evidence indicates that modalities tested in randomised controlled trials (RCTs) are far from 100% applicable and effective.^4^ The guidelines also do not readily apply to the nature and challenges of treating someone with complex PTSD, as opposed to single-event-related PTSD. The oversimplified misrepresentation of the evidence base leads to a constricted provision of services.

In most areas of medicine it is seen that the most severe damage can present the greatest challenges to the intrinsic healing potential of the body. The complexity of the wounding requires an integration of approaches from different specialties and disciplines. This applies not only to the acute interventions but to the painstaking rehabilitation work that may be required over many years to achieve optimal, if still less than full, functioning.

A pain clinic which ignored a history of severe body injuries with functional consequences – and the associated emotional factors – and offered only paracetamol on the grounds that there was good-quality evidence for its analgesic efficacy in many conditions, would fail to treat much intense and prolonged suffering. The psychotherapy often provided for complex PTSD includes no multi-modal approach and those who fail to benefit may then be considered to be treatment-resistant. For patients with complex PTSD the ineffectiveness of psychiatric treatment and subsequent labelling as treatment resistance can then be attributed to an underlying personality disorder, often without adequate assessment or corroboration. Patients who are told that their lack of response to treatment is due to long-standing differences in one or more of the following: their thinking, their emotional regulation, their behaviour, and/or their interpersonal relationships, will feel further invalidation that confirms their inseparable differences from the rest of humanity. If everyone else can learn top-down regulation of distress in response to triggers, the failure to acquire these skills is held to imply deep-seated personality pathology.

## Help-seeking: incentives and disincentives in asking for treatment

There is evidence that even those with non-complex post-traumatic disorders may be reluctant to seek treatment for their condition.^5^ Rates of help-seeking for PTSD are lower than for similar mental disorders such as depression^5^ and studies both internationally and in Northern Ireland have shown extended time to seeking treatment (12–22 years).^6,7^ Reasons given in the USA by those not in treatment who nevertheless recognised that they needed help included a perceived lack of effectiveness (it would not help or did not help in the past), dissatisfaction with services, stigma or fear of forced hospitalisation.^5^ If the wrong treatment paradigm has been offered or the individuals have felt labelled as treatment-resistant or as having a personality disorder, they will be less likely to present again for help and will either not seek any treatment or seek it elsewhere. A narrow approach focused on effortful regulation of distress will deter from contacting services those who believe they have been doing their best to manage their condition themselves.

## Brain stem responses to traumatic experience may be stored and lead to dysfunction

### The body awareness of defence responses

Trauma involving threat, whether physical or social, instigates impulses to defend oneself arising in the midbrain.^8–10^ These impulses may be aborted at an early stage or become active only to be ineffective. They may also be subject to top-down control through the ventromedial prefrontal cortex,^11^ at which level the regulation may also be involuntary. The sequences of movement impulses associated with these responses can be stored ‘in the body’ to the extent that they are outside the ready awareness of working memory and unavailable to access through word-based interventions. Fight, flight, freeze, hide, avoid, attach, submit, despair and uncontrolled activation states have autonomic and motor accompaniments which can be triggered many years after the traumatic episode.^12^ Striatal memory activated preferentially under stress is one mechanism implicating the basal ganglia^13^ in procedural (motor) rather than hippocampal (episodic) memory. Animal studies stress the role of the midbrain periaqueductal gray (PAG) and its connections with the hypothalamus for the full motor and autonomic components of the basic defence responses.^14,15^ It is often through specific motor tension patterns that trauma memories are accessed in body-based psychotherapy: these are the route to healing in somatic experiencing and sensorimotor psychotherapy. This suggests that the midbrain and the basal ganglia loops engaged by defensive movements, actual or thwarted, are instrumental in what is stored in the body following unresolved trauma. The profound impact of sensorimotor psychotherapy and somatic experiencing in survivors of trauma is a source of empirical data which demand thorough evaluation. Rigorous level 2 (case controlled trials, non-randomised) or level 3 (observational studies including surveys) evidence is frequently accepted in medicine when it would be difficult to apply RCT methods, either because of unrealistic statistical power demands or because of a likelihood of harm to patients assigned to a cohort which did not receive the active treatment; for example, the acceptance of psychological first aid after disasters where denying core elements of the approach would be unethical.

### The body awareness of emotions

The animal work of Jaak Panksepp (e.g. Panskeep & Biven^16^) has accumulated over decades into a fundamental realisation that there are basic emotional systems in the mammalian brain. The seven basic emotional systems are: SEEKING/desire; RAGE/anger; FEAR/anxiety; LUST/sexual urges; CARE/maternal nurturance; PANIC/GRIEF/separation distress; and PLAY/physical social-engagement. The midbrain areas involved are the ventral tegmental area for SEEKING and PLAY; the dorsal PAG for RAGE and GRIEF/PANIC; the ventral and dorsal PAG for FEAR and LUST; and the ventral PAG for CARE. All of these basic affective systems are active in humans and are a fundamental part of being human. Many clinical researchers would add shame as a basic affect but the possibility of this being generated in the midbrain is difficult to study in laboratory animals (as discussed in Corrigan^17^). The emotional systems experienced as negative are all activated in various ways by traumatic experience. The aloneness, abandonment and shame of GRIEF/PANIC/separation distress; the terror and dread of FEAR; the explosive energy of anger and RAGE: all of these are commonly encountered in treating PTSD. To treat them as only subcortical disturbances which must be properly managed by the re-trained cortex – or as manifestations of amygdala activation which can be re-learnt through prolonged exposure – is to ignore the fundamental role of emotional response in a person’s interaction with the environment. When the environment is hostile these responses facilitate survival: they are adaptive and based in trends that go far back in brain evolution. In therapy it can be through emotions that healing and lasting transformation are achieved.^18^

## The understanding of the ‘reptilian’ brain as essential for human emotional life

The MacLean description of the triune brain^19^ provides a neat guide to the different levels of the central nervous system in relation to their evolution from organisms more primitive than humans. It is important, however, to appreciate that the ‘reptilian’ brain has evolved in humans to participate in complex functions that would not be available to reptiles. A human may not have as fast a tongue-flick as a lizard, but the human brainstem is supporting behavioural programmes with much greater autonomic and motor variability. A review of neuroimaging studies of the human PAG confirms the involvement of the PAG in many pain syndromes, including fibromyalgia and migraine, and during electroacupuncture.^20^ There are demonstrable PAG responses during emotional experiences such as fear and dread, disappointment, social rejection, hearing aversive sounds, and stressful cognitive tasks. The imaging studies of the human PAG confirm much of what has been found in animal studies.

## What is missing from evidence-based treatments that are not effective?

Patients with complex PTSD who cannot be held in a compassionate and non-judgemental therapeutic relationship will quickly revert to the survival behaviours which have kept them alive. They will default from a therapeutic interaction which carries some of the more threatening features of the ambivalent or disorganised attachment styles to which they may have been exposed from birth. These non-secure relationship templates amplify the impact of later traumatic experiences. Such patients are often exquisitely sensitive at an unconscious level to attachment conflicts. While craving normal attachments, like most humans, any ambivalence or disorganisation in the interactions can lead to an activation of defence response sequences established in early life. The associated survival behaviours will then interfere with the capacity to engage wholeheartedly with the treatments offered. Patients who understand and know the rules to abusive or traumatising interpersonal interaction need to be presented with contingencies to allow them to experience and learn how to have normal, non-traumatising interactions. The attunement with a therapist aware of the importance of attachment in early life experience is essential for the processing of early attachment disruption. The first opportunity some complex trauma patients will have for a stable and non-abusive relationship will be with the therapist. A non-challenging, validating and boundaried therapeutic relationship may be able, eventually, to facilitate the feeling of safety and trust which has been lacking through most, if not all, of the patient’s life.

### The awareness of the body

Those who have disturbing body reactions to triggers reminiscent in some way of the original adverse/traumatic experience will have tried to think their way out of the problem themselves. They may also have been the recipients of common-sense solutions from friends and family. They will have almost certainly discovered the inability to influence through thinking the body sensations of traumatic experience: working memory and cognitive solution areas of the neocortex fail to influence the sensorimotor sequences programmed by the trauma. *Body-Centered Psychotherapy: The Hakomi Method*,^21^ first published in 1990, described mindful body awareness for the elicitation of core material, not necessarily of traumatic origin. This influenced the development of sensorimotor psychotherapy in which the mindful attention to somatic residues of traumatic experience promotes the resolution of these for clinical recovery.^22^ Somatic experiencing was developed by Peter Levine,^23^ whose recent book carries the subtitle ‘How the body releases trauma and restores goodness’.^24^

The extant neurobiology also focuses on the body. For example, van der Kolk,^25^ writing on approaches to the psychobiology of PTSD, included in the title of his chapter the evocative words: ‘the body keeps the score’. Scaer, with a perspective derived from an extensive experience in neurology, concluded that trauma, including preverbal trauma, could leave residues in the body to manifest in later years as clinical syndromes.^26^ Patients with dissociative disorders have difficulty in being in the body experience and becoming safely embodied is a challenge for many.^27^

The gulf between the body-based psychobiology and the talking treatments, evidence-based for PTSD but not for complex PTSD, has been bridged by sensorimotor psychotherapy, somatic experiencing, the Comprehensive Resource Model (CRM),^28^ and other formalised approaches which provide extensive modality-specific training for trauma psychotherapists. Although these are widely used, the lack of RCT data means that they can be readily dismissed if authorities wish to do so: collation of level 2/level 3 evidence would cost much less and set standards for trainers and therapists in the promotion of safe practice. Anecdotally, dropout rate might be the first outcome criterion to employ when empirical studies do evaluate these psychotherapies. Patients who continue to attend because they find sessions helpful and relevant, especially when they have dropped out of other approaches, can provide naturalistic data of empirical value to a caring service.

### The body awareness of safety

The ‘safe place’ is used in the preparation for EMDR to provide an imaginal resource for stabilisation if processing becomes too distressing.^29^ It is also used as a screening tool for EMDR – a patient who cannot access an imaginal place of safety will not readily be offered active reprocessing. This is regarded as an important safeguard as those who have never felt safe have almost certainly suffered from attachment and other trauma from birth, and are likely to be highly dissociative. Calm or peaceful imagery may be used for those who cannot tolerate even the word ‘safe’, but this is fraught with difficulty as the lowering of vigilance may trigger switching to protective ego states or activate trauma-burdened memories. When hypervigilance has long been a default setting, the potential pursuit or creation of a ‘safe place’ or ‘calm place’ may be rejected as too triggering or activating, and alternative creative language will be required. In sensorimotor psychotherapy the attainment of a sense of safety in the body is considered of great importance for stabilisation. This leads to the proposition that it is only when the safe place is sufficiently strong to be experienced at a somatic level that it can be considered to be fully present. Safety resources that do not extend below the cortex are unlikely to have the required depth when processing becomes difficult. Conversely, being able to find the feeling of safety in the body^27^ provides an anchor for processing material which would otherwise be overwhelming. Innovative approaches such as the CRM provide therapists with strategies to build internal resources; thus patients who would otherwise be rejected due to an inability to imagine a safe place can be resourced in alternative ways.^28^

### The understanding of dissociation as essentially neurobiological

Dissociation helps the individual experiencing trauma to survive by compartmentalising the responses to the event. It is then not overwhelming, either neurochemically or physiologically. Peritraumatic dissociation is probably best understood through animal models of stress-induced analgesia to which many neurochemicals contribute (e.g. Ford & Finn^30^). However, it is clear from animal models that, when the trauma involves intense fear, endocannabinoids are released to prevent the overwhelming terror associated with unopposed glutamate, dopamine or acetylcholine transmission in the fear circuits. Riebe *et al*^31^ describe a spill-over effect which triggers the synthesis and release of endogenous cannabinoids. These then bind to presynaptic cannabinoid receptors to down-regulate the release of the fear-promoting neurotransmitters. The endocannabinoid system is active in the fear circuitry of the amygdala, hippocampus and prefrontal cortex, but also in the midbrain PAG where it mediates non-opioid analgesia.^8^ There is evidence that the learning of emotional responses is not confined to the corticolimbic system but occurs also in the midbrain – as would be expected from clinical observations in the treatment of PTSD, such as the resistance of the exaggerated startle response to extinction.

Endogenous opioids promote the analgesia accompanying the passive defence responses mediated by the ventral PAG;^8^ and modulation of these opioids can be used to study behaviour suggestive of terror in laboratory rats.^32^ Lanius^33^ considers endogenous opioids to have a foundational role in dissociative responses to trauma. Whichever chemicals are primarily involved, peritraumatic neurochemical change may contribute longer-term to structural dissociation of the personality.

### The awareness in the body of the level of activation related to trauma

Orientation to the occurrence of a traumatic event precipitates an immediate shift in the body’s level of arousal. For example, being exposed to a direct gaze activates the midbrain in those who are suffering from the after-effects of complex trauma but induces a response at a primarily cortical level in a non-traumatised control group.^34^ This activation readily precipitates a generalised arousal through brainstem nuclei for the release of monoamines and other neurochemicals. From brainstem structures such as the locus coeruleus there are ascending noradrenergic projections to the thalamus and cortex for general arousal as well as downward projections to the spinal cord. Ascending dopaminergic projections from the ventral tegmental area activate the ventral striatum and the substantia nigra. There are major ascending cholinergic and serotoninergic projections from the brainstem. So alerting, arousing, activating stimuli – often involving different appreciation of pain levels – are exerting their influence through deeply subcortical structures.

### The awareness in the body of the residues of attachment response patterns established in infancy

It is particularly easy for those working in the ‘here and now’ to scoff at the idea of working with body feelings left over from experiences of attachment disruption in early life. This is despite there being much description of the relevance of attachment in the development of affect regulation capacities (e.g. Schore^35^) and evidence of the relevance of disorganised caregiving in the development of clinical syndromes (e.g. Lyons-Ruth *et al*,^36^ Hesse *et al*^37^). Attachment disorders can be dismissed as an easy default explanation when there is little evidence of other trauma to explain difficult-to-treat syndromes. However, if the conflicts are approached through body activations brought into awareness while grounded in the experience of specific situations, the patient, rather than the therapist’s model, is leading the enquiry; the body’s response will ground the experience in the ‘here and now’ (Schwenkler, May 2014, personal communication). If there are clear patterns of body response to the present-day relationship conflicts, these are the foundation for identification of cycles of obstruction of the attachment urge, followed by protest, despair, detachment, dissociation and sequences of defence responses. A simple here-and-now trigger, such as disproportionate rage to a partner’s temporary absence, can reveal patterns established in early life. Scaer^26^ sets out the arguments for procedural memory based in brainstem centres being established in infants with preverbal capacities for emotion and sensation. These action tendencies based in procedural memory manifest later as proximity-seeking, social engagement and defensive behaviours,^37^ which may appear at odds with the here-and-now context.

## Healing has no territory

We have argued that the evidence for particular approaches to the psychotherapy of complex PTSD indicates that so-called ‘evidence-based’ modalities – defined as much by those clinical cases excluded as those included – are far from 100% applicable and effective.^4^ Instead, we consider that psychotherapies which acknowledge the role of the somatic residues of traumatic experiences – provide techniques for their resolution – are necessary for the healing of the range of clinical disorders arising from severe and complex traumatic experiences during the brain’s early development. Safety, compassion and patience are needed to counteract the long-term hypervigilance and other threat-based responses, so that the patient is internally resourced and treatment is not quickly rejected. Recognition, and evaluation, of non-RCT but still empirical data from widely used psychotherapies such as sensorimotor psychotherapy and somatic experiencing could widen the evidence base, guiding service development for those suffering in a way which cannot be treated by standard talking therapy.

## A willingness to explore other/additional pathways to healing

Given the limitations of RCT-evidence-based CBT for complex post-traumatic conditions,^4^ it is essential to investigate other approaches consistent with the evolving understanding of the neurobiological underpinnings of traumatic experiences and reactions. In its standard format, EMDR cannot be readily applied in complex post-traumatic disorders but it can have adaptations for use in structural dissociative conditions (e.g. Paulsen^38^). These modifications are often influenced by the many publications (over decades) of hypnotherapy experience of treating complex trauma disorders (e.g. Frederick & McNeal^39^). Moreover, advances in psychotherapy such as Brainspotting^40^ and the CRM^28^ may be effective at a deep level of the psyche because they necessarily involve the midbrain.^41^ Psychotherapies such as sensorimotor psychotherapy and somatic experiencing, which work with emotions and defence responses and access these through awareness of the body and the sensations, movement tendencies and motor impulses ‘remembered’ from the time of the trauma, also work at multiple brain levels. Trauma release exercises^42^ for the discharge of muscular energy residual from adverse events will certainly recruit subcortical areas, as the intrinsic generators of tremor – central oscillators – are not in the neocortex.^43^ Body-oriented breathing exercises stemming from the CRM^28^ and yoga breathing cycles (e.g. Brown & Gebarg^44^), based in the respiratory central pattern generators of the brainstem,^45^ can be used clinically to augment affect regulation.

Russell,^46^ asking why EMDR was not more available to US service personnel, explored the reasons for the dominant treatment paradigms being exclusive. Some of these were financial; some were theoretical or belief-based. Grand^40^ advocates the view that ‘healing has no territory’: developments in therapy should always be encouraged and embraced, although it will inevitably mean that the techniques pioneered will be replaced. For example, the CRM has evolved from resource brainspotting to meet the needs of those individuals with complex trauma and dissociative disorders who require more resourcing than is provided by the safe, attentive and attuned presence of the brainspotting therapist working in a dual attunement frame.^40^ Any important advance will change the field so much that other breakthroughs will follow; each is a temporary way station. No therapeutic paradigm should be allowed to become so dominant that it stifles clinical innovation, especially in the absence of compelling evidence of efficacy for complex disorders.

## Implications for mental health services of fully engaging with the treatment of complex trauma, and research challenges

The high prevalence of trauma exposure and trauma-based disorders with severe consequences for physical and mental health raises the possibility of significant unmet need. Adults who have been exposed to four or more defined categories of adverse childhood experiences have higher risks for alcohol and substance misuse, depression, suicidality and poor physical health.^47^ Childhood sexual abuse increases the risks in adulthood of depression, anxiety, suicidality, alcohol and illicit drug dependence, PTSD symptoms and poor physical health.^48^ The lifetime prevalence of traumatic events and PTSD is high in those with severe mental illness^49^ and there is evidence that trauma therapy can be effective even in this group.^50^ The service implications of the epidemiological findings are that the provision of long-term trauma psychotherapy may be required, and this is expensive. It is considerably cheaper to downplay or ignore the role of trauma and constantly question any psychotherapy methods which have not yet acquired a gold standard RCT evidence base, even if they are expert led or neurobiologically informed. Indeed, services driven by waiting list targets have a disincentive to explore training in, and use of, psychotherapy interventions which require longer therapeutic contact. For dissociative disorders the treatment may require years, even with the best psychotherapy available,^51^ rather than the maximum of 20 sessions currently offered by many services.

If there is no cultural dissociation from the reality of the need for treatment of complex post-traumatic conditions, the consequence would be a caring health service providing treatment for a large number of patients who are only in ill health because they suffered trauma, loss or abuse at an early and critical age. If health service providers recognised the limitations of the RCT evidence base, they could promote training in internationally recognised models and conduct clinical research on those particular psychotherapeutic approaches. Moreover, psychotherapists who are better acquainted with the neurobiological under-pinnings of psychological conditions and their implications for treatment and outcomes, may be less affected themselves by feelings of hopelessness in their long-term clinical endeavours.

NICE^3^ recommended chronic disease management strategies if trials of evidence-based therapies (TF-CBT or EMDR) were ineffective for PTSD; the guidelines did not differentiate the evidence base for PTSD from that for complex PTSD. As we have suggested, these treatments are likely to have been found ineffective for complex PTSD. A focus on sleep hygiene, structured and supported activities as well as coping strategies for chronic problems will neither greatly assist the patient nor allow their therapist the satisfaction of seeing benefits from their skilled and compassionate intervention. Nothing else will be offered if services or systems continue to affectively dissociate from the clinical reality through the blinkered insistence that it is not really happening and that ‘apparently normal’ and ‘getting on with life’ perspectives are the only possible vision.

### Research challenges

For clinical trial research to demonstrate real-world effectiveness of treatments for the range of post-traumatic conditions, the exclusion criteria need to be reduced. Comorbidity with PTSD is the norm, not the exception, yet trials do not reflect this. The measurement of treatment outcomes also needs to reflect more than just any change in PTSD symptoms and to include general functioning, intra- and inter-personal issues, and quality of life. Funding for complex interventions is expensive and a research programme is needed which does not rely on the single intervention for single outcome measure model: this would pose immediate difficulties within a phase-oriented structure for treatment. One intriguing question within the treatment of complex trauma is whether the resourcing required to allow re-processing of trauma experience and memory needs to be provided as a specific phase of treatment prior to any re-processing or whether it can be integral to each therapy session and therefore specific to the issue being reprocessed (as is suggested in the CRM^28^). While discussions about the provision of expensive, innovative medical treatments have occurred, often in public, we are unaware of discussions justifying and limiting the access to long-term psychotherapy for complex disorders.

### Education challenges

The need for outreach and education of referrers should not be underestimated. Evidence from a novel ‘screen and treat’ model after a high-profile terrorist incident showed that despite widespread advertising of the services, general practitioners (GPs) were found to refer few patients to trauma services specifically tasked with assessing and treating individuals in the aftermath of the incident.^52^ There is perhaps even less reason to expect referrals from GPs of individuals with complex post-trauma reactions originating in early development. Within general adult psychiatry the role of trauma may or may not be recognised, largely dependent on the clinician’s interest, knowledge or conceptualisation of cases; it may also perhaps reflect their pessimism about the availability of effective treatment. Potentially significant post-traumatic psychopathology in psychiatric patients^49^ can go unrecognised when there is a failure to include trauma experience in treatment formulations. When this applies to the so-called ‘large T’ trauma causes, there is even less likelihood of the significant attachment disruptions inherent in developmental trauma being identified.

## Conclusions

Within psychological services, the general public have been greatly served by improving access to psychological therapies (IAPT) in England and Wales and similar initiatives elsewhere. However, limited session provision and a dominant therapeutic paradigm that does not approach the needs of patients with chronic, comorbid and complex post-traumatic reactions, leaves those with the most severe symptoms without effective treatment. Patients unable to make use of time-limited cognitive–behavioural strategies may face rejection and labelling, feeling blamed for their non-improvement. In fact, they are victims again, this time of therapists trying to deliver a therapy for a quite different disorder, with managers who expect them to demonstrate consistently improving rating scale scores.

The strategy for the provision of psychological therapies needs to ensure that the most ill are not sidelined and blamed. Current drivers such as waiting list targets are vital to drive access to therapy. However, quality must also be brought to the fore: clinical governance demands the establishment of an environment that allows clinical excellence to thrive; excellence demands that the therapy provided is evidenced for the disorder being treated. In Scotland, there is a strategy for trauma-sensitive services including certain ‘at risk’ groups such as veterans of the armed forces. This is to be applauded. However, clinicians and managers should be educated to clinical need at all points on the trauma spectrum, not just those potentially responsive to CBT or those with combat-related disorders.

Systems that allow long-term trauma psychotherapy rather than time-limited, defined sessional input are needed. Treatment should be influenced by the major developments in affective neuroscience to proceed in a direction that is not affect-phobic. Individuals with highly polysymptomatic post-traumatic conditions, often with more Schneiderian first-rank symptoms than individuals with schizophrenia,^53^ pose major difficulties when monitoring clinical trials. However, the inherent difficulties do not then demand an acceptance – as clinically sufficient – of those techniques validated for the reduction of particular symptoms or symptom clusters within non-complex PTSD. The search for the best treatments for the most traumatised individuals is being hampered by the exclusive acceptance of conditioning, cognitive or emotional learning models which dismiss the fundamental role of affective experience in response to the environment and are, therefore, dehumanising.
